# ADAMTS‐7 Increase Contributes to Worsened Neurological Outcome in Mice on High Fat Diet

**DOI:** 10.1002/cns.70877

**Published:** 2026-04-12

**Authors:** Yang Xu, Jun Li, Zhiyi Zuo

**Affiliations:** ^1^ Department of Anesthesiology University of Virginia Charlottesville Virginia USA

**Keywords:** a disintegrin and metalloproteinase‐7, cerebrovascular remodeling, high fat diet, ischemic brain injury

## Abstract

**Background:**

Obesity and associated metabolic disturbance are associated with worse outcomes after brain ischemia. Cerebrovascular remodeling mediated by matrix metalloproteinase 9 (MMP‐9) may contribute to this poor outcome. A disintegrin and metalloproteinase‐7 (ADAMTS‐7) has been shown to regulate vascular remodeling in peripheral vessels. This study was designed to determine the role of ADAMTS‐7 in regulating MMP‐9 activity, cerebrovascular remodeling and neurological outcomes in mice.

**Methods:**

Six‐week‐old CD‐1 male mice were fed regular diet (RD) or high fat diet (HFD) for 10 weeks before they had left middle cerebral arterial occlusion (MCAO) for 1.5 h. Their brains were harvested for Western blotting, immunostaining or cerebral vascular casting. Lentiviral particles containing shRNA targeting ADAMTS‐7 mRNA or scramble shRNA were injected intracerebroventricularly.

**Results:**

HFD feeding increased ADAMTS‐7 and MMP‐9 activity, reduced middle cerebral arterial root diameter, and worsened neurological outcomes after the MCAO. Silencing ADAMTS‐7 attenuated these HFD‐induced effects. ADAMTS‐7 was expressed in the neurons, astrocytes, oligodendrocytes, and blood vessels in the brain.

**Conclusions:**

HFD increased ADAMTS‐7 to lead to MMP‐9 activation, which then induces cerebrovascular remodeling and poor neurological outcomes after brain ischemia. ADAMTS‐7 is expressed in multiple cell types in the brain.

## Graphical abstract

High fat diet (HFD) increases a disintegrin and metalloproteinase‐7 (ADAMTS‐7) in the brain of mice with and without ischemia. Increased ADAMTS‐7 reduces tissue inhibitor of metalloproteinase‐1 (TIMP‐1) to increase matrix metalloproteinase 9 (MMP‐9) activity. These effects contribute to the HFD‐induced cerebrovascular remodeling and worsened neurological outcomes after brain ischemia and reperfusion (I/R) in mice fed HFD.

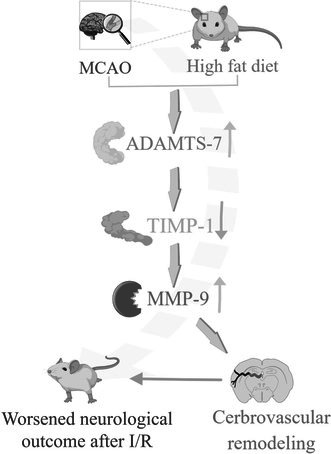

## 1 Introduction

Ischemic stroke is a leading cause of mortality and morbidity in the United States and the world [1]. Obesity and the associated metabolic disturbance are major risk factors for ischemic stroke and contributors to poor outcomes after the stroke [2]. High fat diet (HFD) intake is considered a cause of increased incidence of obesity, especially in young patients, in the United States [3, 4, 5].

Our previous study has shown that HFD feeding induces obesity and hyperlipidemia in young adult mice. The mice develop cerebrovascular remodeling via increased matrix metalloproteinase 9 (MMP‐9) activity, and this vascular remodeling contributes to the worsened neurological outcome after focal brain ischemia [6]. However, it is not clear how HFD increases MMP‐9 activity in the brain.

A disintegrin and metalloproteinase‐7 (ADAMTS‐7) has been found to be involved in vascular remodeling and the development of atherosclerotic plaque in the peripheral vessels [7, 8, 9]. This effect may be mediated by enhancing the degradation of tissue inhibitor of metalloproteinase‐1 (TIMP‐1) that is known to inhibit MMP‐9 [8]. Thrombospondin 1 (TSP‐1) is a known binding protein of ADAMTS‐7 [9] and can decrease TIMP‐1 [10]. Thus, ADAMTS‐7 is an upstream protein that can increase TSP‐1 and decrease TIMP‐1 to increase MMP‐9. These effects have been shown in the peripheral tissues but not in the brain yet. The expression of ADAMTS‐7 protein in the brain is not known. The role of ADAMTS‐7 in the HFD‐induced cerebrovascular remodeling is not reported.

This study aimed to determine (1) the role of ADAMTS‐7 in HFD‐induced increase of MMP‐9 activity, cerebrovascular remodeling and poor outcomes after brain ischemia, (2) the involvement of TSP‐1 and TIMP‐1 in the role of ADAMTS‐7 in the HFD effects, and (3) the cell types expressing ADAMTS‐7 in the brain. To achieve these determinations, mice were fed a regular diet (RD) or HFD for 10 weeks. ADAMTS‐7 was silenced by shRNA in the brain. Middle cerebral arterial occlusion (MCAO) was performed to induce transient focal brain ischemia.

## 2 Materials and Methods

### 2.1 Mice

Male CD‐1 mice, aged 6 weeks, were purchased from Charles River and housed in a specific pathogen‐free facility under a 12‐h light/dark cycle, with free access to food and water. They were then randomized to have HFD (45% of calories supplied by fat; D12451, Research Diets Inc., New Brunswick, NJ) for 10 weeks or continued to be on RD (4.5% of calories supplied by fat; Harland Laboratories, Dublin, VA) prior to being used in the experiments. Mice fed HFD and RD were then randomized to different experimental groups in each set of experiments. All animal experiments adhered to the NIH Guide for the Care and Use of Laboratory Animals and were approved by the University of Virginia Institutional Animal Care and Use Committee. Blinding could not be effectively executed because the mice on HFD were obese compared with mice on RD, but blinding principles were followed in groups of mice on the same diet. The assessments of neurological outcomes and biochemical assays were objective.

### 2.2 Middle Cerebral Artery Occlusion (MCAO)

Transient focal brain ischemia was induced via intraluminal occlusion of the left middle cerebral artery (MCA) for 90 min as we did before [11]. Mice were anesthetized with 1.5% isoflurane in O2. A 7–0 nylon monofilament with a silicone‐rubber coated tip (tip diameter: 0.21 mm, coating length: 3–4 mm; #1622, Beijing CiNongtech Co. Ltd., Beijing, China) was inserted into the internal carotid artery and advanced to the origin of the MCA. The animals were allowed to wake. They were re‐anesthetized briefly by isoflurane at 90 min after the onset of MCAO to remove the nylon monofilament. Throughout the surgical procedure, rectal temperature was maintained at 37°C ± 0.5°C using a heating pad. Sham‐operated animals underwent identical anesthesia and surgical procedures, except for the MCAO.

### 2.3 Determination of Infarct Volume

At 24 h post‐MCAO, mice were anesthetized with 1.5% isoflurane. The brains were dissected and sliced into five coronal sections, which were incubated in 2% 2,3,5‐triphenyltetrazolium chloride (catalogue number: T8877‐50G, Sigma‐Aldrich) for 15 min at 37.0°C. Following incubation, the slices were immersion‐fixed in 4% paraformaldehyde. Infarcted and non‐infarcted hemispheres were then analyzed using Image J software (National Institutes of Health, USA).

### 2.4 Rotarod Test

The rotarod test was used to assess post‐stroke motor deficits. In brief, mice were placed on a rotating rod that accelerated from 4 to 40 rpm over the course of 5 min. The time at which the mouse fell off the rod (latency to fall) was recorded. Each mouse underwent three trials 2 days before, 1 day before, and on the day of surgery to establish a pre‐MCAO baseline. Mice were tested 24 h after the MCAO in three trials with 15 min intervals. The average of these 3 trials was divided by the average of the 3 trials just before the MCAO.

### 2.5 Western Blotting

Proteins were isolated from the peri‐infarct region of mice 24 h after the MCAO or from the corresponding brain region of mice without MCAO. Samples were incubated with primary antibodies against ADAMTS‐7 (1:1000, catalogue number: 26836, Proteintech), TIMP‐1 (1:1000, catalogue number: B100‐74551, Novusbio), TSP‐1 (1:1000, catalogue number: 18304–1‐AP, Proteintech) or glyceraldehyde 3‐phosphate dehydrogenase (GAPDH, catalogue number: 5174, Cell Signaling Technology) (1:1000) overnight at 4°C. Samples were then incubated with horseradish peroxidase‐linked anti‐rabbit (1:10,000, catalogue number: SC2357, Santa Cruz) or anti‐mouse (1:10,000, catalogue number: SC516102, Santa Cruz) secondary antibodies for 1 h at room temperature. Protein bands were visualized using a sensitivity substrate kit (catalogue number: 34096, Thermo Scientific) and analyzed using ImageJ software. Band values of other proteins were normalized to those of the GAPDH levels in the same samples.

### 2.6 Immunohistochemistry

Mice were deeply anesthetized and transcardially perfused with 0.9% NaCl, followed by 4% paraformaldehyde in phosphate buffered saline (PBS) 24 h after the MCAO. Brains were harvested and cryoprotected in 30% sucrose in PBS, and frozen serial coronal brain sections (25 μm thick) were prepared using a Precisionary CF‐6100 cryostat. Sections were blocked with 5% donkey serum in PBS for 1 h, followed by overnight incubation at 4°C with primary antibodies against ADAMTS‐7 (1:100, catalogue number: 26836, Proteintech), TIMP1 (1:100, catalogue number: B100‐74551, Novusbio), TSP‐1 (1:100, catalogue number: MA5‐13398, Invitrogen), glial fibrillary acidic protein (GFAP) (1:100, catalogue number: ab53554, Abcam), ionized calcium‐binding adaptor molecule 1 (Iba1) (1:100, catalogue number: ab5076, Abcam), neuronal nuclei (NeuN) (1:100, catalogue number: 66836–1, Proteintech), oligodendrocyte transcription factor 2 (Olig2) (1:300, catalogue number: 35988, Cell Signaling Technology), myelin basic protein (MBP) (1:300, catalogue number: 66003–1‐Ig, Proteintech) and cluster of differentiation 31 (CD31) (1:200, catalogue number: AF3628, R&D Systems). After being washed, sections were incubated for 1 h at room temperature with secondary antibodies anti‐mouse IgG (Alexa Fluor 594, A21203, Invitrogen), anti‐rabbit IgG (Alexa Fluor 594, catalogue number: A21207, Invitrogen) or anti‐goat IgG (Alexa Fluor 488, catalogue number: A11055, Invitrogen). Sections were then mounted and cover‐slipped with Hoechst (1:10000, catalogue number: 33342, Thermo Scientific). Fluorescence images were captured with an inverted Zeiss LSM 700 fluorescence microscope and analyzed as we did before [12, 13]. Briefly, immunofluorescence images were acquired using identical microscope settings across all groups and analyzed with ImageJ. Background signals were excluded by subtracting channel specific thresholds to each image. Co‐localization was defined as the spatial overlap of positive pixels between the two channels. The degree of co‐localization of two biomarkers was calculated as the percentage of positive area for both biomarkers in the total image area. For each sample, data from 6 randomly selected fields in the brain regions were averaged to reflect the level of co‐localization prior to statistical analysis.

### 2.7 Nissl Staining

To determine the infarct brain volume 14 days after the MCAO, formaldehyde‐fixed brains harvested at this delayed time point were cut into 26 μm sections and subjected to Nissl staining. The sections were sequentially immersed in 100%, 95%, and 80% ethanol for 30 s each, followed by staining with Cresyl Violet Solution (catalogue number: C0775, Sigma) for 2 min. After rinsing in distilled water, the sections were dehydrated through a graded alcohol series, cleared in xylene and mounted with neutral resin. In this study, the stained sections were scanned, and the infarct areas were measured using Image J software. These procedures were performed as we did previously [14].

### 2.8 Cerebral Vasculature Casting

As we did before [6], a 20% solution of freshly prepared vascular polyurethane casting resin (1:1 by weight/volume, Specialty Resin) was mixed with blue dye. Brains were removed and fixed in 4% paraformaldehyde in PBS at 4°C overnight. Stereomicroscopic images of the whole brain were taken from the bottom to measure the interior width of the middle cerebral artery root. At least six mice from each group were analyzed to derive the final data.

### 2.9 MMP‐9 Gelatin Zymography Assay

The samples harvested 24 h after the MCAO were separated using Novex 10% Zymogram Plus Gels (catalogue number: 24080291, Invitrogen). Following electrophoresis, the gel was washed with zymogram renaturing buffer (catalogue number: LC2670, Novex) and then incubated overnight at 37°C in zymogram developing buffer (catalogue number: LC2671, Novex). The gel was stained with the colloidal blue staining kit (catalogue number: LC6025, Invitrogen) for 4 h to visualize the bands. Gelatinolytic activity appeared as clear areas in the gel. The gels were photographed and quantitatively analyzed as we did before [6].

### 2.10 Intracerebroventricular Injection

The injection was performed as we described before [15]. ADAMTS‐7 shRNA lentiviral particles shRNA (catalogue number: sc‐140,869‐V, Santa Cruz) and scramble shRNA lentiviral particles (catalogue number: sc‐108,080, Santa Cruz) were at 1.0 × 10^6^ infectious units in 200 μL. Each mouse received 2 μL viral particles. The stereotactic intracerebroventricular injection site was 0.3 mm posterior, 1.0 mm lateral and 2.3 mm deep relative to the bregma. shRNA was administered intracerebroventricularly 4 weeks prior to the induction of MCAO.

### 2.11 Statistical Analysis

Data of all animals that lived to the predetermined end of observation times were included in the analysis. No other specific inclusion and exclusion criteria were set before the experiments or during the experiments. Parametric results in normal distribution are presented as mean ± S. E. M. (*n* ≥ 6). The data were analyzed by *t*‐test or one‐way analysis of variance followed by the Tukey test because all data from this study were normally distributed. Survival curve was analyzed by the Logrank test. Differences were considered significant at *p* < 0.05 based on two‐tailed hypothesis testing. All statistical analyses were performed with GraphPad Prism 8.0.

## 3 Results

The body weights of CD‐1 mice fed HFD for 10 weeks were heavier than those fed RD (33.7 ± 0.9 g for RD‐fed mice, 51.5 ± 1.6 g for HFD‐fed mice, *n* = 6, *p* < 0.0001). These results suggest that HFD induces obesity. The body weights of mice fed HFD and receiving shRNA targeting ADAMTS‐7 or scramble shRNA were 49.5 ± 2.2 g or 48.8 ± 1.7 g, respectively (*n* = 6), which were not different from those of mice fed HFD. These results suggest that receiving shRNA does not affect the body weights and possibly the obesity of these mice.

### 3.1 HFD Increased ADAMTS‐7 and Induced Cerebrovascular Remodeling, and Silencing ADAMTS‐7 Attenuated HFD‐Induced Cerebrovascular Remodeling and MMP‐9 Activity Increase

**FIGURE 1:**
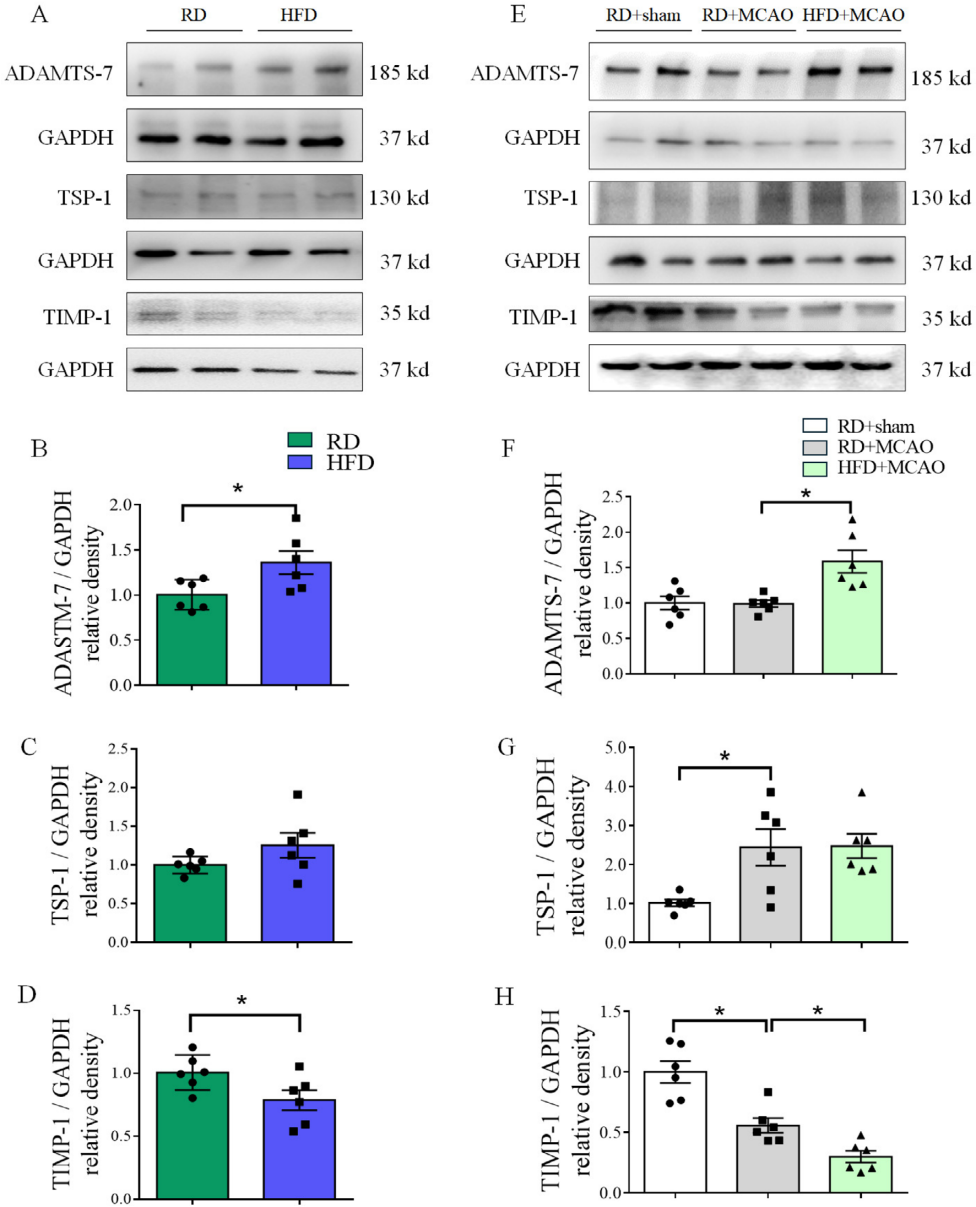
HFD increased ADAMTS‐7 and TIMP‐1. (A) Representative images of Western blots of mice without MCAO. (B) Abundance of ADAMTS‐7 in mice without MCAO. (C) Abundance of TSP‐1 in mice without MCAO. (D) Abundance of TIMP‐1 in mice without MCAO. (E) Representative images of Western blots of mice with MCAO. (F) Abundance of ADAMTS‐7 in mice with MCAO. (G) Abundance of TSP‐1 in mice with MCAO. (H) Abundance of TIMP‐1 in mice with MCAO. Results are mean ± SEM (*n* = 6). * *p* < 0.05.

As the first step to indicate the role of ADAMTS‐7 in the HFD effects on the brain, the expression of ADAMTS‐7 in the brain was determined. HFD feeding for 10 weeks increased ADAMTS‐7 and reduced TIMP‐1 in mice (Figure 1A–D). These differences between mice fed HFD and RD remained 24 h after the MCAO (Figure 1E–H). MCAO increased TSP‐1 and TIMP‐1 but did not affect the amount of ADAMTS‐7 (Figure 1A–H). These results suggest that HFD increases ADAMTS‐7 and reduces TIMP‐1 in mice with or without the MCAO.

**FIGURE 2:**
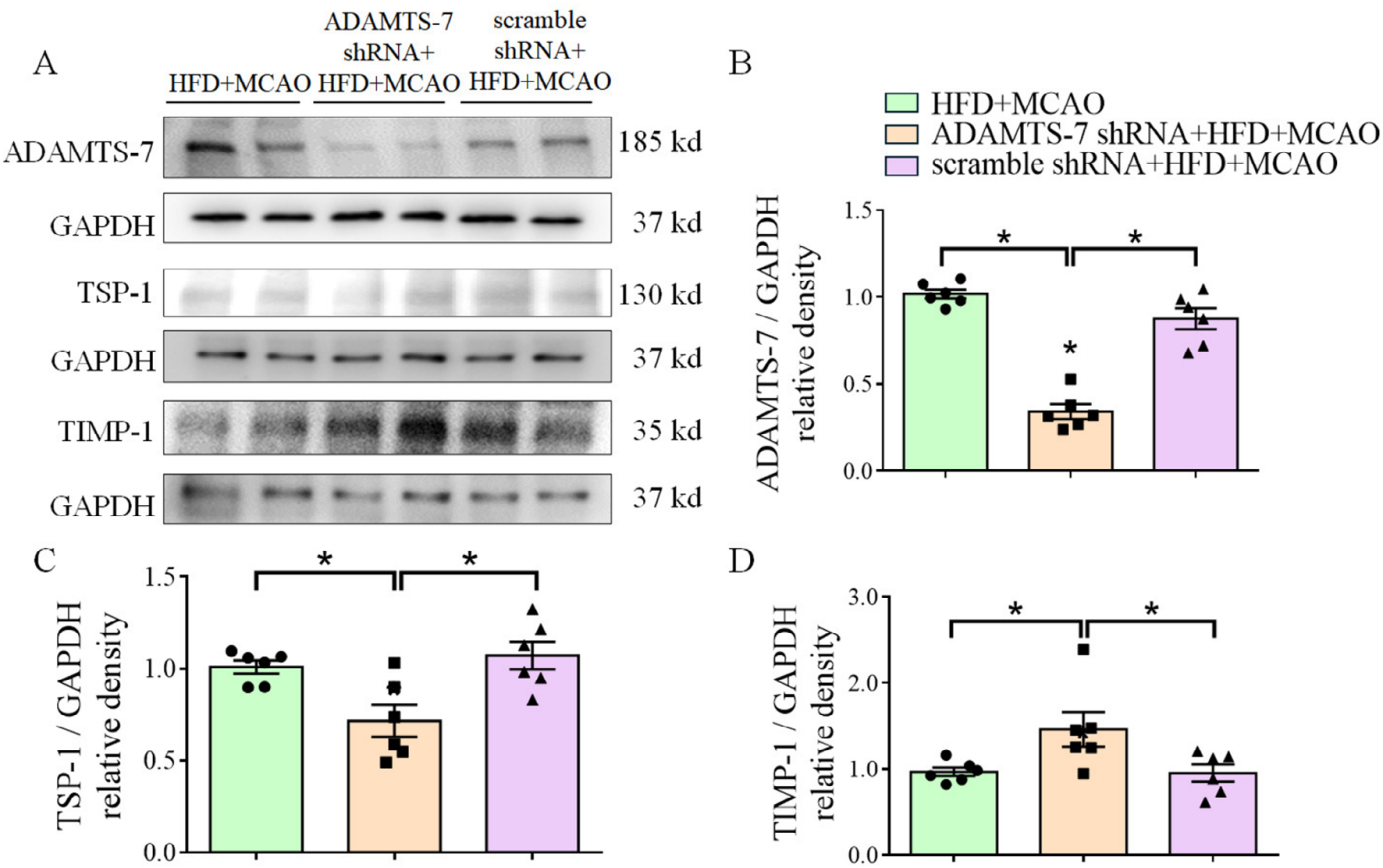
Silencing ADAMTS‐7 decreased the expression of TSP‐1 and TIMP‐1 in mice fed HFD. (A) Representative images of Western blots. (B) Abundance of ADAMTS‐7. (C) Abundance of TSP‐1. (D) Abundance of TIMP‐1. Results are mean ± SEM (*n* = 6). * *p* < 0.05.

**FIGURE 3:**
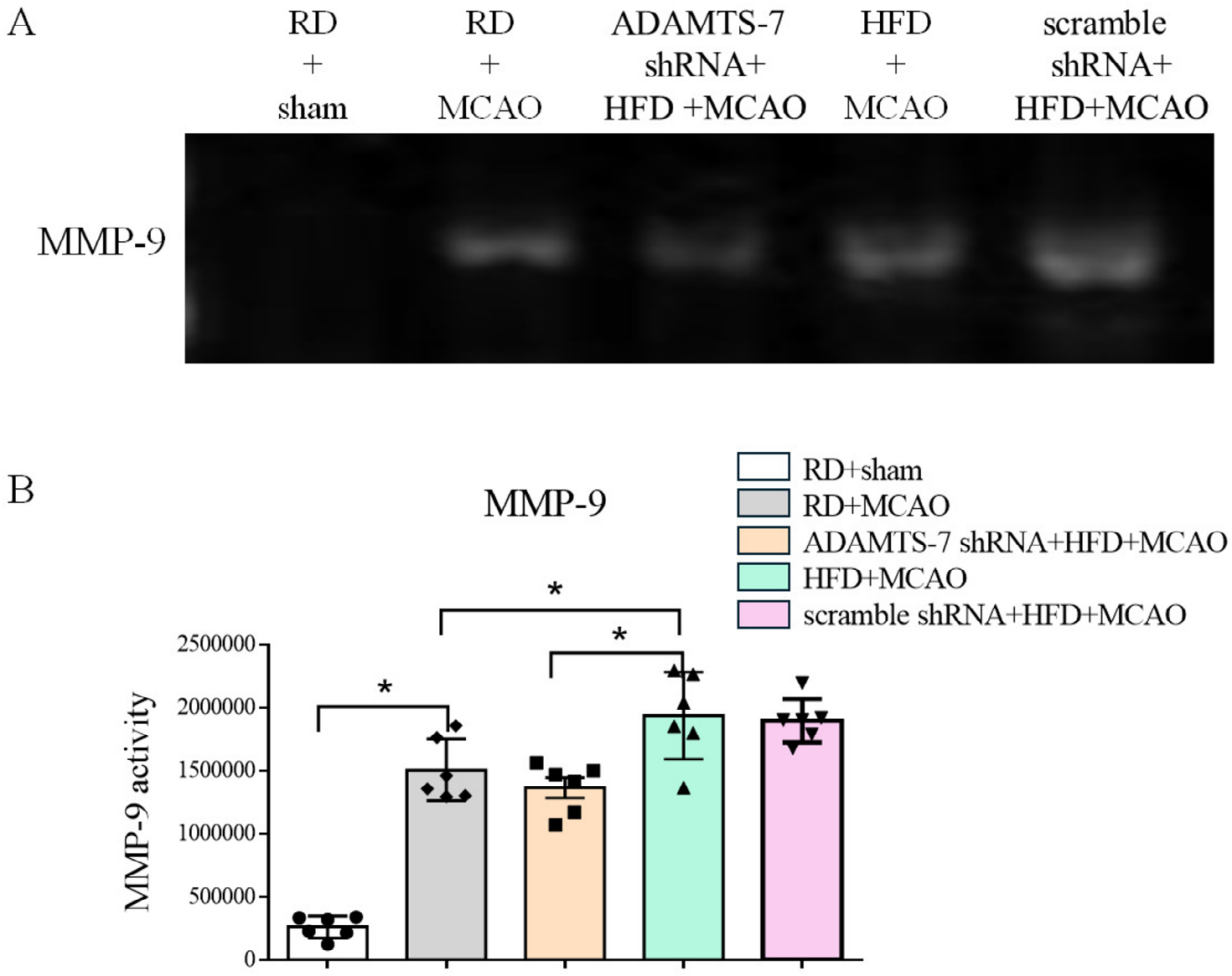
Silencing ADAMTS‐7 decreased MMP‐9 activity. (A) Representative images of zymographic gel. (B) MMP‐9 activity. Results are mean ± SEM (*n* = 6). E.M. * *p* < 0.05.

**FIGURE 4:**
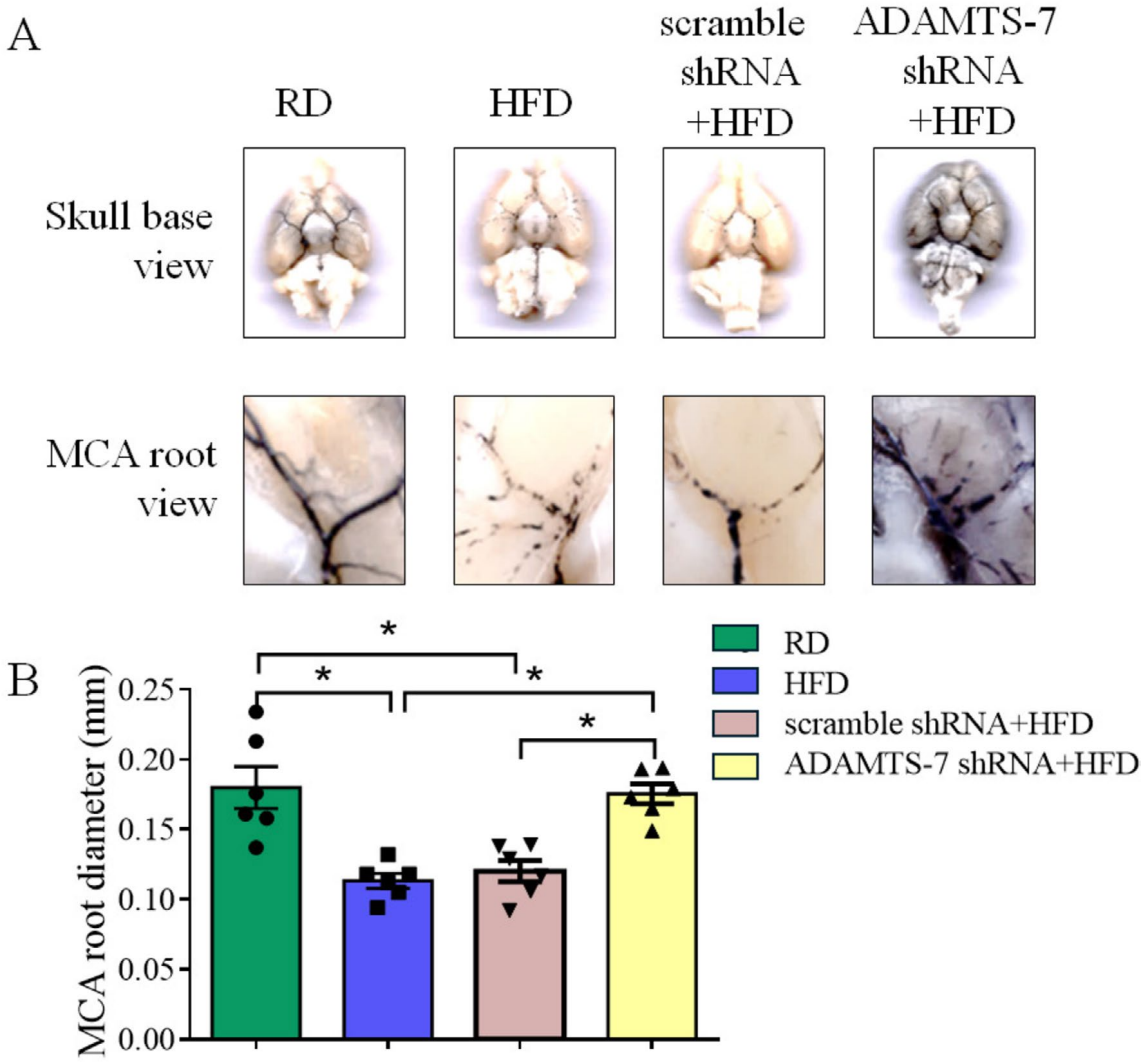
Silencing ADAMTS‐7 attenuated HFD‐induced cerebrovascular remodeling. (A) Representative images of brain. (B) Middle cerebral arterial diameter. Results are mean ± SEM (*n* = 6). * *p* < 0.05.

To further determine the role of ADAMTS‐7 in the ischemic brain injury of mice fed HFD, we investigated whether ADAMTS‐7 was involved in the MMP‐9‐mediated cerebrovascular remodeling in mice fed HFD. This remodeling is considered a contributing factor for the worsened neurological outcomes after a focal brain ischemia in these mice [6]. First, the shRNA targeting ADAMTS‐7 but not the scramble shRNA reduced the expression of ADAMTS‐7. Silencing ADAMTS‐7 decreased TSP‐1 and increased TIMP‐1 (Figure 2). Second, mice fed HFD had an increased MMP‐9 activity in the brain tissues, and silencing ADAMTS‐7 reduced MMP‐9 activity in these mice (Figure 3). Finally, consistent with what we have reported previously [6], HFD reduced the diameter of MCA root (Figure 4), suggesting that HFD induced cerebrovascular remodeling. Silencing ADAMTS‐7 attenuated the reduction of the MCA root diameter in mice fed HFD (Figure 4). These results suggest that increased ADAMTS‐7 contributes to the HFD‐induced cerebrovascular remodeling.

### 3.2 Silencing ADAMTS‐7 Improved Neurological Outcomes in HFD‐Fed Mice

**FIGURE 5:**
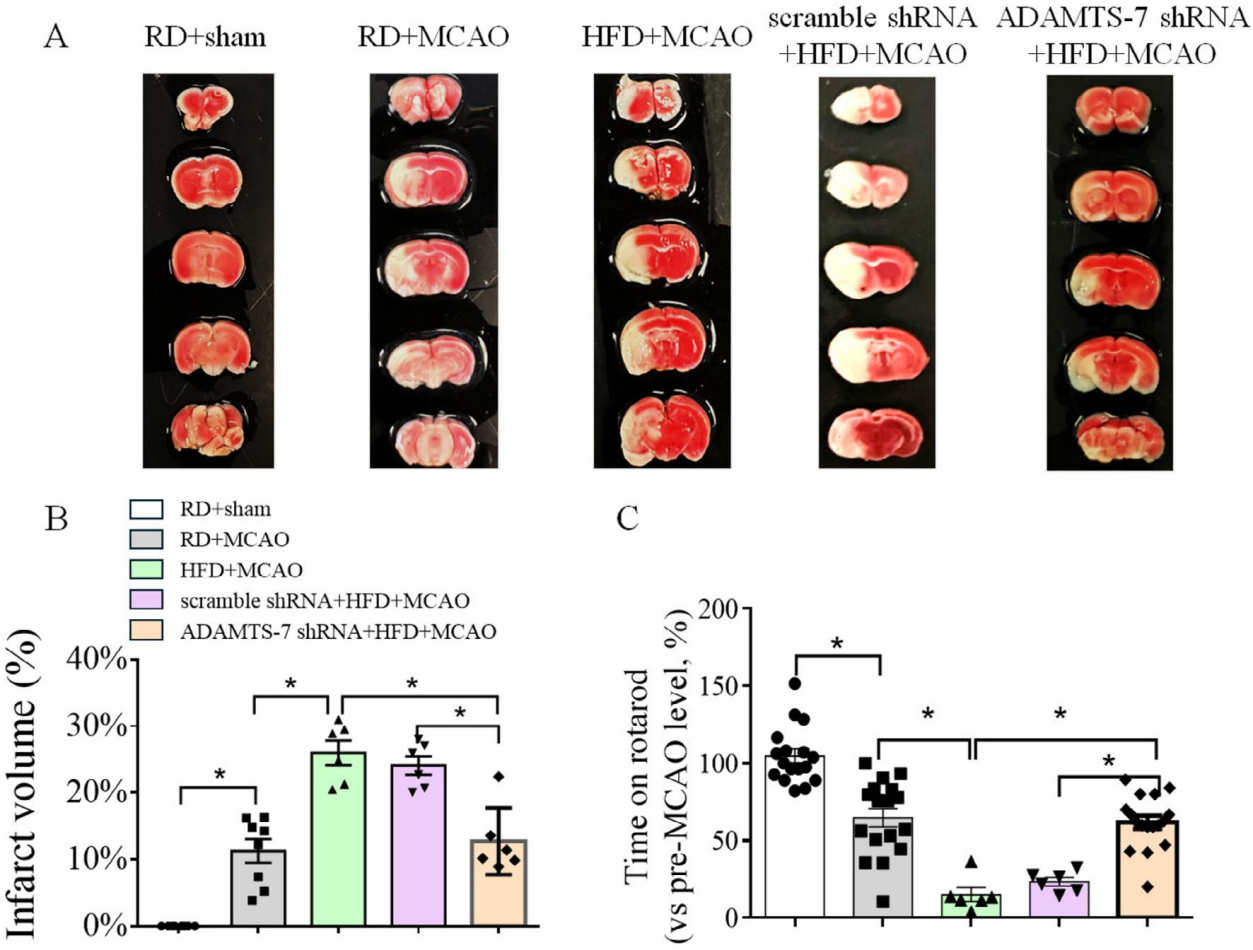
HFD worsened neurological outcomes and silencing ADAMTS‐7 expression improved neurological outcomes in mice fed HFD. (A) Representative images of brain slices. (B) Infarct brain volumes. (C) Performance on rotarod. Results are mean ± SEM (*n* = 6–17). * *p* < 0.05.

To determine whether ADAMTS‐7 was involved in the worsened ischemic brain injury in mice fed HFD, mice with or without ADAMTS‐7 silencing had a MCAO to induce focal brain ischemia. Mice on HFD for 10 weeks had a higher infarct brain volume and poorer performance on rotarod than mice on RD 24 h after the MCAO. Silencing ADAMTS‐7 reduced the infarct brain volume and improved performance on rotarod of the mice fed HFD (Figure 5A–C). These results suggest that HFD feeding worsens neurological outcome after transient focal brain ischemia and that this worsened outcome is attenuated by silencing ADAMTS‐7.

**FIGURE 6:**
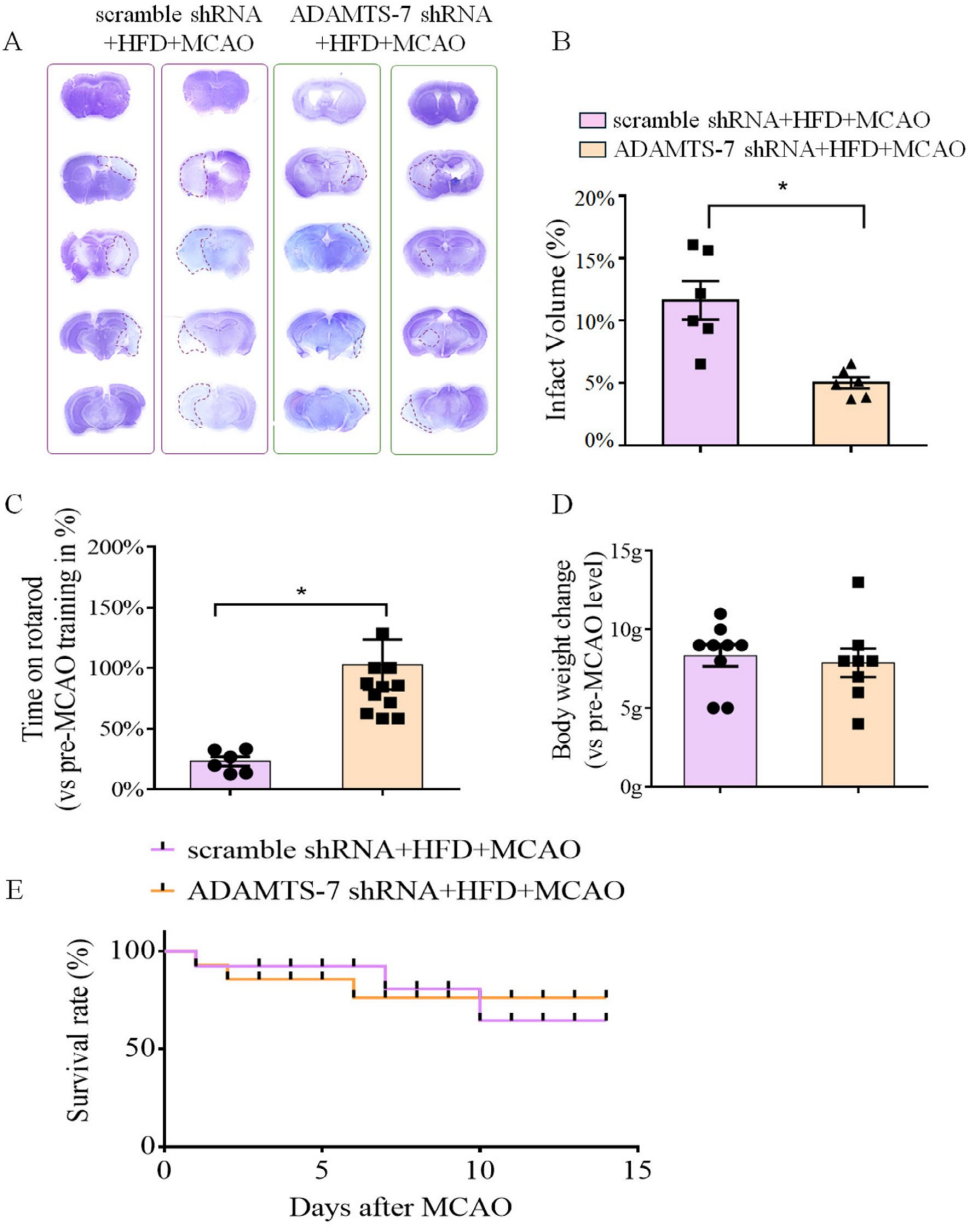
ADAMTS‐7 expression silencing improved long‐term neurological outcomes. (A) Representative images of brain sections. (B) Infarct volumes. (C) Performance on rotarod. (D) Body weight reduction after the MCAO. (E) Survival curve. Results are mean ± SEM (*n* = 6–11). M. * *p* < 0.05.

Similar to the results assessed at 24 h after the MCAO, silencing ADAMTS‐7 also reduced infarct brain volume and improved performance on rotarod of mice fed HFD when the assessment was performed 14 days after the MCAO (Figure 6A–C). These results suggest that silencing ADAMTS‐7 improves long‐term neurological outcomes in these mice. However, there was no difference in the decrease of body weights between HFD‐fed mice with and without ADAMTS‐7 silencing at 14 days after the MCAO compared with their corresponding body weights before the MCAO (Figure 6D). There was no difference in the survival curve between the mice with or without ADAMTS‐7 silencing during the 14 days of observation (Figure 6E). These findings indicate that ADAMTS‐7 silencing in the brain does not affect the mortality and the general well‐being of mice after the MCAO.

### 3.3 ADAMTS‐7 Was Co‐Localized With TSP‐1 and TIMP‐1 and Was Expressed in Neurons, Astrocytes and Oligodendrocytes but Not in Microglia

**FIGURE 7:**
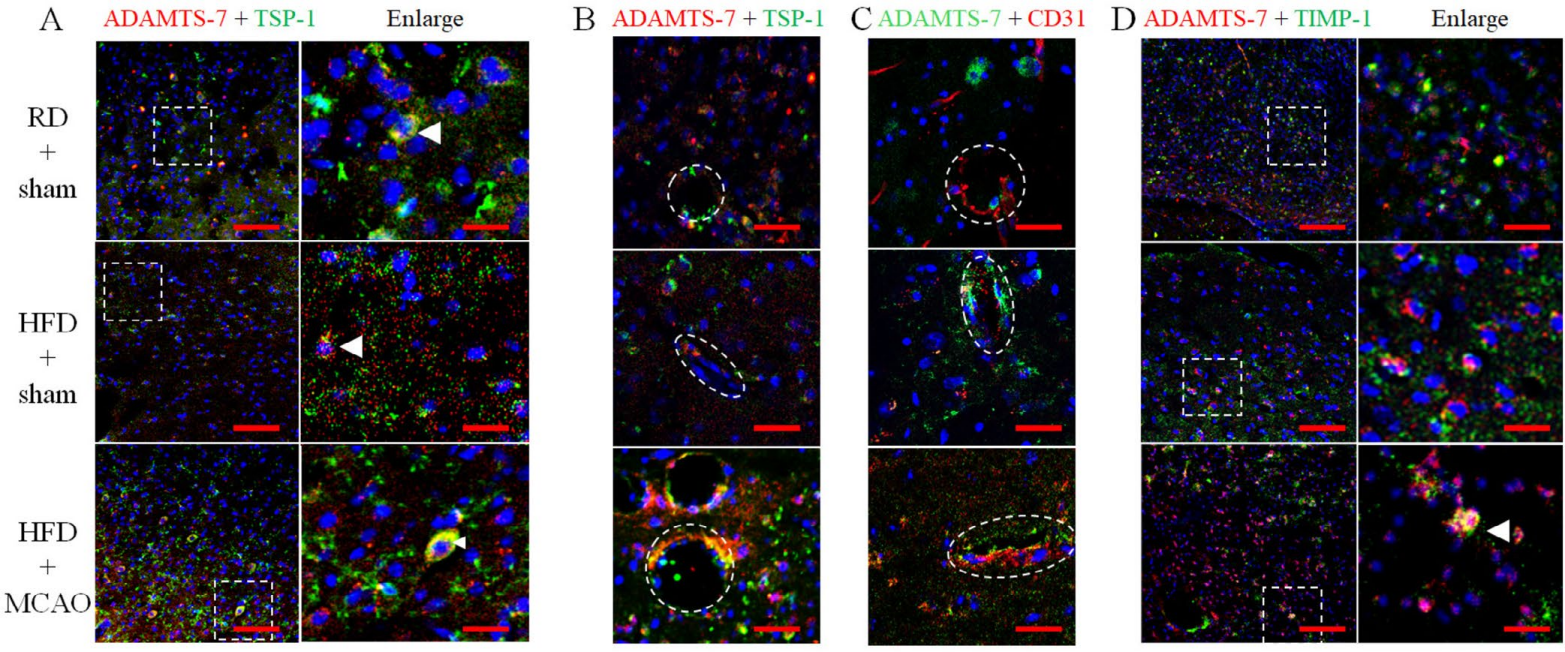
Co‐staining of ADAMTS‐7 with TSP‐1 and TIMP‐1 in the frontal cerebral cortex area 1. (A) Co‐staining of ADAMTS‐7 with TSP‐1. (B) Co‐staining of ADAMTS‐7 with TSP‐1 in blood vessel appearance images. (C) Co‐staining of ADAMTS‐7 with CD31. (D) Co‐staining of ADAMTS‐7 with TIMP‐1. The right panels in panels A and D are the enlarged images of the areas indicated in the left panels. Scale bar = 100 μm for the left panel and = 20 μm for the right panel in panels A and D, = 40 μm for panels B and C.

To determine whether ADAMTS‐7 was co‐localized with its downstream proteins in the brain, immunostaining was performed. The ADAMTS‐7 was co‐localized with TSP‐1 in mice with and without MCAO in the brain (Figure 7A). These two proteins were expressed in the blood vessels (Figure 7B,C). Similarly, ADAMTS‐7 staining was co‐localized with TIMP‐1 staining (Figure 7D).

**FIGURE 8:**
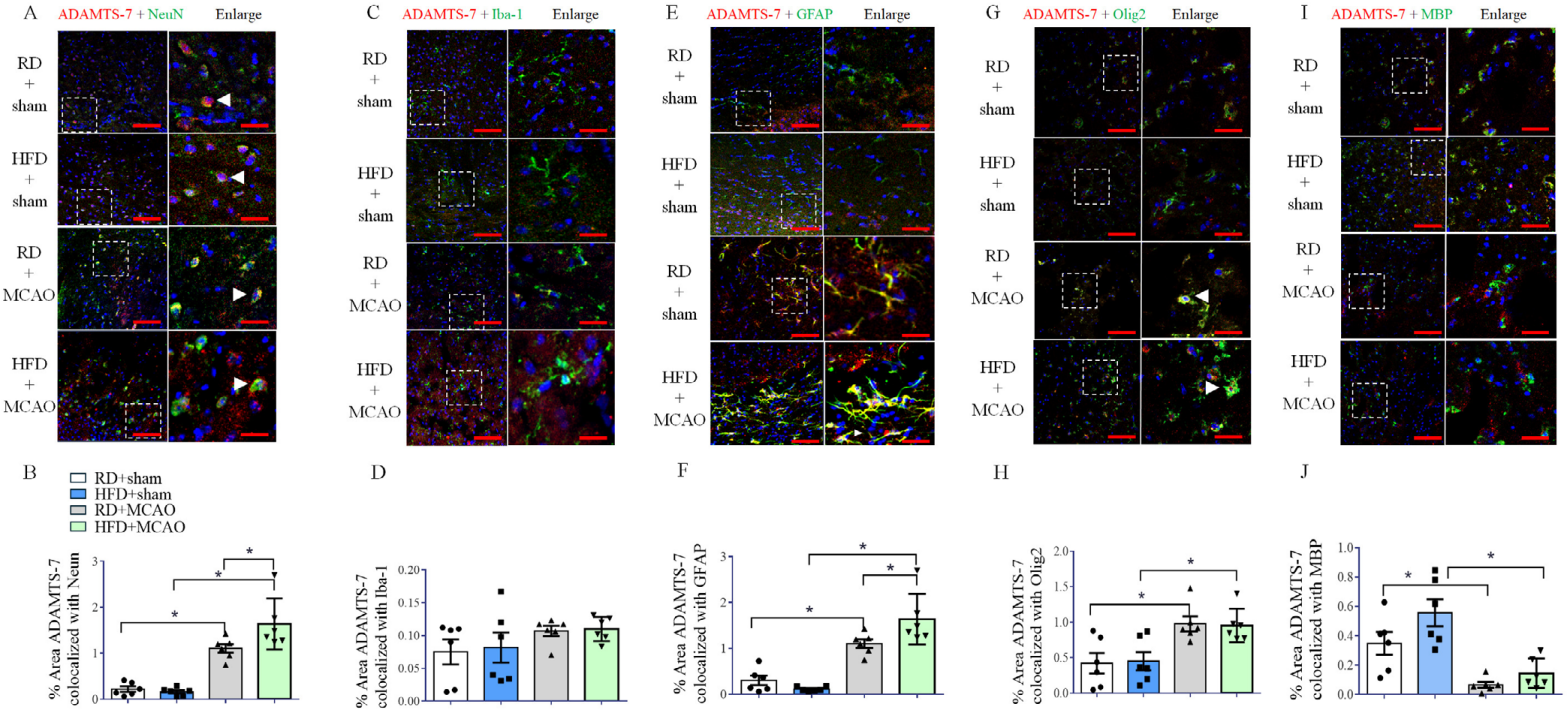
Co‐staining of ADAMTS‐7 with NeuN, GFAP, Olig2 and MBP in the cerebral cortex. (A) Co‐staining of ADAMTS‐7 with NeuN. (B) Quantification of co‐staining of ADAMTS‐7 with NeuN. (C) Staining of ADAMTS‐7 and Iba‐1. (D) Quantification of co‐staining of ADAMTS‐7 with Iba‐1. (E) Co‐staining of ADAMTS‐7 with GFAP. (F) Quantification of co‐staining of ADAMTS‐7 with GFAP. (G) Co‐staining of ADAMTS‐7 with Olig2. (H) Quantification of co‐staining of ADAMTS‐7 with Olig2. (I) Co‐staining of ADAMTS‐7 with MBP. (J) Quantification of co‐staining of ADAMTS‐7 with MBP. The right panels in panels A, C, E, G and I are the enlarged images of the areas indicated in the left panels. Scale bar = 100 μm for the left panel and = 20 μm for the right panel. Results are mean ± SEM (*n* = 6). * *p* < 0.05.

To identify which cells expressed ADAMTS‐7, cell specific markers were stained. ADAMTS‐7 was expressed in cells that were positive for NeuN (Figure 8A), a protein marker for neurons [16]. This co‐localization was increased in mice with MCAO, and the mice fed HFD and with MCAO had the highest co‐localization (Figure 8B). There was no obvious co‐localization of ADAMTS‐7 and Iba‐1 (Figure 8C,D), a microglial biomarker [16], in mice fed RD or HFD. The co‐localization of ADAMTS‐7 staining with the staining of GFAP, an astrocytic biomarker [16], was not obvious in the brain of mice fed RD or HFD but without brain ischemia. However, the co‐localization of ADAMTS‐7 and GFAP was increased in mice with MCAO. This co‐localization was the highest in the HFD‐fed mice with MCAO (Figure 8E,F). Similarly, there was co‐localization between ADAMTS‐7 and Olig2, a biomarker for all oligodendrocytes of the entire lineage including early progenitor cells to mature oligodendrocytes [17, 18]. This co‐localization was increased by MCAO in both RD‐ and HFD‐fed mice (Figure 8G,H). There was also co‐localization between ADAMTS‐7 and MBP, a biomarker for mature oligodendrocytes [17, 18]. This co‐localization was decreased by MCAO in both RD and HFD‐fed mice (Figure 8I,J). These results suggest that ADAMTS‐7 is expressed in the neurons, astrocytes and oligodendrocytes, and that the expression of ADAMTS‐7 is increased in the neurons and astrocytes of mice with brain ischemia, especially in those fed HFD. Brain ischemia increases ADAMTS‐7 in oligodendrocytes when all oligodendrocytes are considered but decreases it in mature oligodendrocytes.

## 4 Discussion

Consistent with our previous studies [6, 7, 8, 9, 10, 11, 12, 13, 14, 15, 16, 17, 18, 19, 20], HFD feeding led to cerebrovascular remodeling, MMP‐9 activity increase, and poor neurological outcomes after brain ischemia in this study. Silencing ADAMTS‐7 attenuated these HFD‐induced effects. HFD also increased ADAMTS‐7 in the brain. These results indicate the role of ADAMTS‐7 in HFD‐induced detrimental effects on the brain.

Our previous study has shown that MMP‐9 activity increase contributes to the HFD‐induced cerebrovascular remodeling and poor neurological outcomes after brain ischemia [6]. ADAMTS‐7 can increase the degradation of TIMP‐1 to reduce the inhibition of TIMP‐1 on MMP‐9 activity [8]. Studies have shown that ADAMTS‐7 can bind with TSP‐1 and can reduce TIMP‐1 [9, 10]. Thus, HFD‐induced increase of ADAMTS‐7 may contribute to the MMP‐9 activity increase in these mice. Consistent with this possibility, silencing ADAMTS‐7 increased TIMP‐1 and decreased MMP‐9 activity, suggesting that the effects of ADAMTS‐7 are mediated by TIMP‐1. Silencing ADAMTS‐7 also reduced TSP‐1, indicating a possible role of TSP‐1 in mediating the effects of ADAMTS‐7 on TIMP‐1 and MMP‐9. However, our results did not show that HFD‐fed mice had a higher level of TSP‐1 than RD‐fed mice. In addition, a previous study has shown that ADAMTS‐7 can enhance the degradation of TSP‐1 [9]. This effect may contribute to the finding that HFD feeding increases ADAMTS‐7 but does not increase TSP‐1 expression in our study. Taking together all findings from our study, it is not clear whether TSP‐1 plays a role in the effects of ADAMTS‐7 on MMP‐9 activity in HFD‐fed mice. Nevertheless, our results support the ADAMTS‐7/TIMP‐1/MMP‐9 pathway in the HFD‐induced cerebrovascular remodeling and worsened ischemic brain injury.

ADAMTS‐7 has been shown to be associated with unstable phenotypes in human carotid and coronary arterial atherosclerosis [7, 8, 9, 10, 11, 12, 13, 14, 15, 16, 17, 18, 19, 20, 21] and to inhibit re‐endothelialization of injured arteries [9]. However, the expression of ADAMTS‐7 protein in the brain tissues is not known. Our results showed that ADAMTS‐7 was expressed in the neurons, astrocytes, oligodendrocytes, and blood vessels in the brain. The expression of ADAMTS‐7 in the neurons and astrocytes of ischemic penumbral brain tissues was significantly increased in mice fed HFD and with MCAO compared with the expression of ADAMTS‐7 in the non‐ischemic brain tissues of mice fed HFD and in the ischemic brain tissues of mice fed RD. ADAMTS‐7 staining was co‐localized with TSP‐1 and TIMP‐1 staining, consistent with the previous findings that ADAMTS‐7 can bind TSP‐1 and TIMP‐1 [8, 9]. These protein expression results and the results of silencing ADAMTS‐7 specifically in the brain indicate that ADAMTS‐7‐mediated cerebrovascular remodeling and poor neurological outcomes in HFD‐fed mice are due to ADAMTS‐7 increase in the brain and may not be from the peripheral cells circulating to the brain.

ADAMTS‐4 has been shown to contribute to the HFD‐induced atherosclerosis and plaque instability in mouse aorta [22]. Our results add evidence for the involvement of another ADAMTS member, ADAMTS‐7, in mediating HFD‐induced effects on the brain vessels. These findings indicate the important role of ADAMTSs in the effects of HFD on blood vessels.

Our results show that ADAMTS‐7 is expressed in the neurons, astrocytes, and oligodendrocytes. This expression is increased in the ischemic brain tissues. Associated with this increase, the MMP‐9 activity was increased, especially in the ischemic brain tissues of HFD‐fed mice. MMP‐9 can impair blood–brain barrier [23], which is a known pathophysiological process for ischemic brain injury [24]. Thus, in addition to the cerebrovascular remodeling that can work through various mechanisms to affect ischemic brain injury, such as delayed recovery of blood reperfusion to the ischemic brain tissues after the opening of occluded blood vessels [20], other mechanisms including disruption of blood–brain barrier may contribute to the worsened ischemic brain injury in HFD‐fed mice. Interestingly, brain ischemia increases ADAMTS‐7 in oligodendrocytes when all oligodendrocytes (from early progenitor stages to mature phase) are considered but decreases ADAMTS‐7 in mature oligodendrocytes. This finding presents a complex nature of the regulation of ADAMTS‐7 in the ischemic brain tissues. Future studies are needed to understand the physiological and pathophysiological functions of ADAMTS‐7 in different types of brain cells, such as astrocytic activation and neuronal injury after brain ischemia or other stresses.

Our findings may have significant implications. Diet‐induced obesity and metabolic disturbance are common. Our study has shown that ADAMTS‐7 is a target for reducing HFD‐induced detrimental effects in the brain. Interventions specifically inhibiting ADAMTS‐7 or increasing TIMP‐1 in the brain may be developed to block these effects. Also, ADAMTS‐7 mRNA is detected in the brain [25]. Our study shows that ADAMTS‐7 protein is expressed in the brain cells and blood vessels and that its expression in the neurons and astrocytes is increased in the ischemic penumbral tissues. These results may support further investigation of the biological and pathological effects of ADAMTS‐7 in the brain. For example, ADAMTS‐7 can upregulate tumor necrosis factor α and interleukin 17, proinflammatory factors [26, 27]. This proinflammatory effect may play an important role in inflammatory diseases, such as arthritis [26, 27]. Thus, the role of ADAMTS‐7 in the inflammatory process in the brain can be further investigated. Since inflammation is involved in the process of many diseases and HFD can induce inflammation in the brain [3], ADAMTS‐7 may affect the progress of these diseases and the effects of HFD on the brain via its regulation of neuroinflammation. Specifically, neuroinflammation is a known major contributor to ischemic brain injury [28, 29]. The increased ADAMTS‐7 may lead to increased neuroinflammation, in addition to the induced cerebrovascular remodeling and impaired blood–brain barrier, for the worsened neurological outcomes in HFD‐fed mice after brain ischemia. Future comprehensive studies will determine the details of the involvement of neuroinflammation in the effects of ADAMTS‐7 on the ischemic brain.

Our study has limitations. First, we showed that ADAMTS‐7 expression was increased in mice fed HFD. We have not investigated how HFD increases ADAMTS‐7. Second, we have identified that ADAMTS‐7 can be expressed in many types of cells. We have not determined that ADAMTS‐7 from which cell types is critical for HFD‐induced detrimental effects on the brain. However, ADAMTS‐7 can be a secreted protein [27], and ADAMTS‐7 in the extracellular space may be the major form to inhibit MMP‐9. In this case, ADAMTS‐7 from all cell types contributes to the effects of HFD on the brain. Finally, our previous studies on HFD‐induced cerebrovascular remodeling and worsening ischemic brain injury were all performed in male animals [6, 7, 8, 9, 10, 11, 12, 13, 14, 15, 16, 17, 18, 19, 20]. In addition, the estrous cycle in mice is about 4 to 5 days. Inducing brain ischemia in the same estrous cycle phase in all mice in one set of experiment is challenging. Thus, only male mice were used in this study. For this reason, our current findings cannot be generalized to female mice.

In summary, we have shown that HFD induces cerebrovascular remodeling and poor neurological outcomes after brain ischemia. These effects may be mediated by ADAMTS‐7 increase. ADAMTS‐7 is expressed in the neurons, astrocytes, oligodendrocytes, and blood vessels in the brain.

## Funding

This work was supported by University of Virginia, The Robert M. Epstein Professorship endowment. National Institutes of Health, R01 NS099118. School of Medicine, University of Virginia, Department of Anesthesiology.

## Ethics Statement

Not a human study. The animal protocol was approved by the Institutional Animal Care and Use Committee of the University of Virginia (Charlottesville, VA, USA) (protocol number: 3114, to Zhiyi Zuo's laboratory).

## Consent

The authors have nothing to report.

## Conflicts of Interest

The authors declare no conflicts of interest.

## Data Availability

The data that support the findings of this study are available from the corresponding author upon reasonable request.
